# Role of Vitamin-E on Rat Liver-amiodarone: An Ultrastructural Study

**DOI:** 10.4103/1319-3767.48967

**Published:** 2009-04

**Authors:** Mohamed Samir A. Zaki, Refaat A. Eid

**Affiliations:** Department of Anatomy, Faculty of Medicine, King Khalid University, Abha, Saudi Arabia; 1 Department of Pathology (Electron Microscopy Unit), Faculty of Medicine, King Khalid University, Abha, Saudi Arabia

**Keywords:** Vitamin-E, rat liver, amiodarone, ultrastructural study

## Abstract

**Background/Aim:**

Amiodarone, a class III antiarrhythmic drug, has been found to be effective in the management of patients with life-threatening ventricular arrhythmias. The aim of this study was to test whether the co administration of vitamin-E with amiodarone can reduce amiodarone-induced liver damage.

**Materials and Methods:**

Twelve male albino rats were divided into three groups (ml vegetable oil/day by oral gavages daily for 2 weeks and were used as control group. The rats of the second group received 5.4 mg amiodarone/100 gm rat dissolved in vegetable oil daily by oral gavages for 2 weeks. In the third group, the rats received 5.4 mg amiodarone and 5 mg vitamin-E/100 gram rat dissolved in 2 ml vegetable oil by oral gavages daily for 2 weeks. Two weeks after treatment, the rats were sacrificed and liver specimens were immediately taken and processed for transmission electron microscopic examinations.

**Results:**

Sections from the rat liver receiving amiodarone examined by electron microscopy showed disrupted hepatocytes with increased vacuolations. Degenerated organelles and disrupted nuclei were observed. The microvilli of bile canaliculi were disrupted and the hepatocytes showed increased lipid contents. Both endothelial cells and Kupffer cells were damaged. Phospholipids inside the mitochondria showed a loss of cristae. Sections from the liver of rats received amiodarone and vitamin-E showed lesser effects, especially in depositions of phospholipids in the mitochondria and the whole organelles and the nucleus showed minor damage in comparison to the previous group.

**Conclusion:**

Milder hepatotoxic effects are seen in rats administered amiodarone and vitamin E simultaneously suggesting that vitamin-E may play a role in amelioration of the effects of amiodarone.

Amiodarone is an efficacious antiarrhythmic agent that is limited clinically by numerous adverse effects.[1] In addition, amiodarone, and its primary metabolite, desethylamiodarone (DEA), show a previously unrecognized antioxidant activity on human lipoprotein oxidation.[2] Amiodarone has been shown to generate free radicals that may be involved in the pathogenesis of its toxicity[1,3,4] and in its cellular immunity decreasing effect.[5] However, Sarma ***et al***[6] and Bolt ***et al***,[7] found that oxidative stress is not involved in the pathogenesis of amiodarone toxicity. The potential mechanisms of amiodarone toxicity include direct cytotoxicity in the form of lysosomal phospholipidosis development as well as an indirect effect through membrane destabilization.[8,9] Amiodarone has been shown to produce microvesicular steatosis of the liver in some recipients. Also, the effects of amiodarone on the mitochondrial oxidation of fatty acids in mice were determined.[10] Characteristic lamellar lysosomal inclusion bodies representing phospholipidosis were found in two of the 14 specimens studied ultrastructurally.[11]

The antioxidant vitamin-E was shown to reduce lysosomal phospholipidosis[12] and amiodarone toxicity.[13] Vitamin-E significantly inhibited the formation of the thiobarbituric acid reactive substance, which is used as a measure of free radical mediated-lipid peroxidation in tissue homogenates.[14]

The aim of this study was to test whether the co-administration of vitamin-E with amiodarone can reduce amiodarone-induced liver damage using the electron microscope.

## MATERIALS AND METHODS

### Amiodarone treatment

Twelve male albino rats were divided into three groups of four rats each. In the first group, the rats received 2 ml vegetable oil/day by oral gavages daily for 2 weeks and were used as a control group. The rats of the second group received 5.4 mg amiodarone (chlorhydrate D') from Sanofi, France; /100 gm dissolved in vegetable oil daily by oral gavages for 2 weeks. This corresponds to the maximum human daily therapeutic dose converted into the equivalent rat dose according to Paget's table.[15] In the third group, the rats received 5.4 mg amiodarone and 5 mg vitamin E/100 (α-tocopherol acetate) gm dissolved in 2 ml vegetable oil by oral gavages daily for 2 weeks. The dose of vitamin E was chosen as an effective antioxidant dose in rats according to Calfee-Mason ***et al***.[16]

### Preparation of liver tissue for electron microscopy

Small pieces of the liver parenchyma were fixed in 2.5% glutaraldehyde for 24 hours. The small pieces were washed by phosphate buffer (0.1 M, pH 7.4). Postfixation was made in 1% osmium tetroxide buffered to pH 7.4 with 0.1 M phosphate buffer at 4°C for 1-2 h and then washed again in phosphate buffer to remove the excess fixative. The samples were dehydrated through ascending grades of ethanol followed by clearing in propylene oxide. The specimens were embedded in araldite. Polymerization was obtained by placing the capsules at 60°C. Ultrathin sections (100 nm) were prepared using ultramicrotome and picked up on uncoated copper grids. Following double staining with uranyl acetate and lead citrate, sections were examined and photographed using a JEOL 100 Cx transmission electron microscope, Japan.[17]

### Data analysis

Malondialdehyde (MDA) was determined and measured according to the method of Yoshioka ***et al***,[18] using a spectrophotometer (Milton Roy 3000 ARRAY double beam spectrophotometer; USA) and subjected to statistics according to the methods of Aherene and Dannil.[19] Data were entered to an epi-info file using epi-info version 6.02 software computer packages.[20] Data were expressed as mean ± SD and the remaining significance used was one way analysis of variance (ANOVA) to compare several means. The levels of significance were < 0.05 and < 0.001.

## RESULTS

### Group I (Control group)

**Figure 1:**
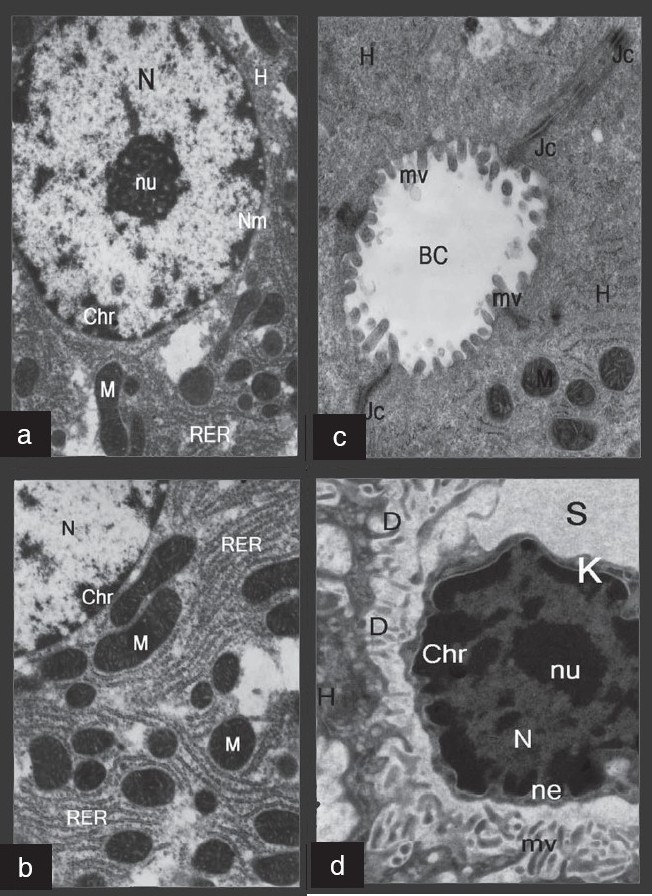
Electron micrographs of normal liver. (a): A hepatocyte (H) showing mitochondria (M), rough endoplasmic reticulum (RER) and a nucleus (N) that is surrounded by a nuclear membrane (Nm) with chromatin masses (Chr) and nucleolus (nu). (b): A higher magnification of the previous figure showing a hepatocyte with mitochondria (M) that contains cristae, and a plenty of rough endoplasmic reticulum (RER). A part of the nucleus (N) is also seen. (c): A hepatocyte (H) with bile canaliculus (BC) that shows microvilli (mv) and junctional complexes (Jc) are seen in this figure. (d): A hepatocyte with blood sinusoid (S): the space of Disse (D) contains Kupffer cells (K), with its nucleus that have a nuclear envelope (ne), chromatin masses (Chr) and nucleolus (nu). A hepatocyte (H) with microvilli (mv) is also seen

On electron microscope examination of the sections from the rat liver of the control group showed a hepatocyte with mitochondria with prominent cristae, plenty of rough endoplasmic reticulum and a nucleus that was surrounded by a nuclear membrane with chromatin masses and a nucleolus [Figure 1a, Figure 1b]. The mitochondria of the hepatocytes showed prominent cristae. In addition, these hepatocytes showed a bile canaliculus that contains microvilli and junctional complexes [Figure 1c]. A hepatocyte with microvilli, blood sinusoid, space of Disse that contains Kupffer cells with its nucleus having a nuclear envelope, chromatin masses and nucleolus were seen [Figure 1d].

### Group II (Rats receiving amiodarone only)

**Figure 2:**
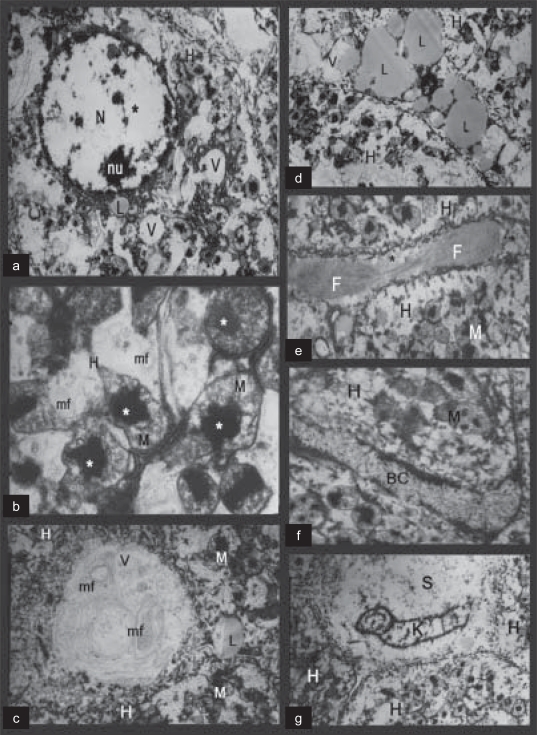
Electron micrographs of amiodarone-treated rats. (a): A degenerated hepatocyte (H) with many vacuoles (V), the damaged nuclear chromatin (star) and a nucleolus (nu) is seen. (b): A damaged hepatocyte with mitochondria (M) that have lipid deposits (stars). Myelin figures (mf) are also seen. (c): A degenerated hepatocyte (H) with vacuoles (V) that contains myelin figures (mf). Lipid droplets (L) and mitochondria (M) are also present. (d): A degenerated hepatocyte (H) with many lipid droplets (L) and vacuoles (V) are shown. (e): A degenerated hepatocyte (H) with dilated intercellular space (star) that contains collagen fibrils (F). Degenerated mitochondria (M) are also seen. (f): A degenerated hepatocyte (H) with dilated bile canaliculus (BC) and abnormal microvilli is seen. (g): A degenerated hepatocyte (H) with fragmented blood sinusoid (S) and destructed Kupffer cells (K)

The fine structure from rats receiving amiodarone drug showed degenerated hepatocytes with many vacuoles and damaged nuclear chromatin [Figure 2a, Figure 2c]. The mitochondria showed lipid deposits. Membranous structures arranged in whorled arrays (myelin figures) and vacuoles in the degenerated hepatocytes were also seen [Figure 2b, Figure 2c]. Many lipid droplets were also shown as scattered in the cytoplasm [Figure 2d]. In addition, the hepatocytes show a dilated intercellular space that contains collagen fibrils [Figure 2e], with the bile canaliculus being dilated and showing abnormal microvilli [Figure 2f]. The Kupffer cells were destructed and the blood sinusoids were fragmented [Figure 2g].

### Group III (Rats receiving amiodarone and vitamin E)

**Figure 3:**
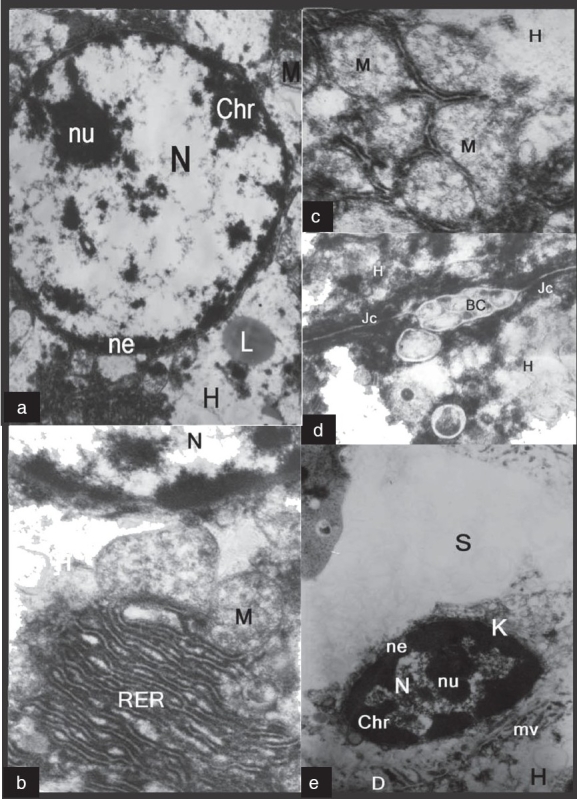
Electron micrographs of amiodarone- and vitamin E-treated rats. (a): A hepatocyte (H) showing rounded nucleus (N) with an intact nuclear envelope (ne), chromatin masses (Chr), nucleolus (nu) and minimal lipid droplet (L). (b): A hepatocyte (H) revealing intact rough endoplasmic reticulum (RER), nucleus (N) and damaged mitochondria (M). (c): A hepatocyte (H) showing damaged mitochondria (M) without deposits. (d): A hepatocyte (H) with normal bile canaliculus (BC) that shows normal microvilli and junctional complex (Jc) is seen. (e): A hepatocyte (H) showinga blood sinusoid with intact Kupffer cell (K) that shows the nucleus (N) surrounded by a nuclear envelope (ne) and chromatin masses (Chr) plus nucleolus (nu) and microvilli (mv)

On electron microscopic study of a section in the hepatocytes from rats received amiodarone and vitamin E showed rounded nuclei with intact nuclear envelope, chromatin masses, nucleolus and minimal lipid droplet [Figure 3a]. The hepatocytes of the same group revealed intact rough endoplasmic reticulum but the mitochondria were still damaged, but without phospholipids deposits [Figure 3b, Figure 3c]. In addition, the hepatocytes showed normal bile canaliculi with normal microvilli and intact junctional complexes [Figure 3d]. The blood sinusoid of the hepatocytes was intact with a healthy Kupffer cell. The nuclei of the hepatocytes were intact and surrounded by a nuclear envelope and chromatin masses plus nucleolus were also shown [Figure 3e].

### Statistical analysis

There was a significant increase in plasma MDA in group II as compared to group I [Table 1], (***P*** < 0.001).

**Table 1:** Comparison between group II and group I

Data	Group II Mean ± SD	Group I Mean ± SD	T	P
Plasma MDA	17.67 ± 2.55	7.40 ± 1.45	7.01	< 0.001

There was a nonsignificant increase in plasma MDA in the group III as compared with group I [Table 2], (***P*** > 0.05).

**Table 2:** Comparison between group III and group I

Data	Group III Mean ± SD	Group I Mean ± SD	T	P
Plasma MDA	8.23 ± 1.71	7.40 ± 1.45	0.735	> 0.05

There was a significant increase in plasma MDA in the group III as compared with group II [Table 3], (***P*** < 0.001).

**Table 3:** Comparison between group II and group III

Data	Group II Mean ± SD	Group III Mean ± SD	T	P
Plasma MDA	17.67 ± 2.55	8.23 ± 1.71	6.16	< 0.001

Analysis of variance (F-Test) showed a significant difference between the studied groups [Table 4], (***P*** < 0.001).

**Table 4:** Analysis of variance (F-test) of plasma MDA in the studied data

Data	Group I Mean ± SD	Group II Mean ± SD	Group III Mean ± SD	F	P
Plasma MDA	7.4 ± 1.45	17.67 ± 2.55	8.23 ± 1.71	33.96	< 0.001

## DISCUSSION

Amiodarone is a lipophilic antiarrhythmic/antianginal drug which is able to influence the physicochemical status of biological lipid components. Since oxidation of lipids is affected by their physicochemical state and amiodarone binds to lipoprotein. Lapenna ***et al***,[2] hypothesized that the drug may exert an antioxidant activity on human lipoprotein oxidation.

The fine structure of the hepatocytes from the rats that received amiodarone drug showed a degenerated hepatocyte with many vacuoles with damaged nuclear chromatin. These necrotic hepatocytes with disrupted cytoplasm and a large number of pathological organelles suggest the occurrence of amiodarone toxicity.[21] Membranous structures arranged in whorled arrays (myelin figures) were suggestive of lysosomal phospholipidosis. These myelin figure containing lysosomes were oval or irregular in shape and vacuoles in the degenerated hepatocytes were also seen. Lewis ***et al***,[11] demonstrated that phospholipidosis appears to be a generalized systemic effect of cationic amphophilic compounds, such as amiodarone. Moreover, an electron microscopic study of liver tissue done by Rigas ***et al***,[22] showed phospholipid-laden lysosomal lamellar bodies, suggesting that both toxic and hypersensitivity, liver injury can occur in response to amiodarone. The presence of phospholipid-laden lysosomal lamellar bodies may help differentiate amiodarone hepatotoxicity from alcoholic liver disease or other causes of hepatic steatosis. In addition, Guigui, ***et al***,[23] illustrated that phospholipidosis could be only be a morphological marker of intrahepatic accumulation of the drug.

The mitochondria showed deposits and many lipid droplets. These intramitochondrial lipids are characterized by the lack of limiting membrane, amorphous appearance, a medium to high density, and a rounded or irregular form.[24] In addition, mitochondrial alterations and free radicals have been implicated in the etiology of amiodarone induced toxicities.[25] Moreover, Fromenty ***et al***,[10] concluded that amiodarone inhibits the mitochondrial beta-oxidation of fatty acids and produces microvesicular steatosis of the liver. Moreover, the hepatocytes show a dilated intercellular space that contains collagen fibrils and the bile canaliculus were dilated and showed abnormal microvilli. Ballooning of hepatocytes, Mallory bodies, and fibrosis were also common. Lewis ***et al***,[11] demonstrated some changes included in the nucleus and characteristic lamellar lysosomal inclusion bodies representing phospholipidos.

In our study, the Kupffer cells were destructed with the blood sinusoids that were found to be fragmented. This is in accordance with the study of Ireton[26] who found ultrastructurally, numerous cytoplasmic inclusions with a membranous or lamellar structure identical to those described in phospholipidosis were the most striking features seen in hepatocytes, biliary epithelial cells, Kupffer cells, and endothelial cells. Electron microscopy done by Kannan ***et al***,[27] revealed the presence of lipid inclusion bodies in the liver, lung, and alveolar macrophages of desethylaminodarone-treated rats.

In the present investigation, the electron microscopic study of a section in the liver from rats received amiodarone and vitamin-E showed a rounded nucleus with an intact nuclear envelope, chromatin masses, nucleolus and minimal lipid droplets. The hepatocytes of the same group revealed an intact rough endoplasmic reticulum but the mitochondria were still damaged without deposits. In addition, the hepatocytes showed a normal bile canalicului with normal microvilli and intact junctional complexes. The blood sinusoid of the hepatocytes was intact with a healthy Kupffer cell. The nuclei of the hepatocytes were intact and surrounded by a nuclear envelope and chromatin masses plus nucleolus were also shown. Vitamin E served to improve the antioxidant defense system.[28]

It has been demonstrated by several authors that antioxidants, such as vitamin-E[12,27] and the flavonoid-type silibinin,[3] when coadministered with amiodarone reduce lysosomal phospholipidosis. However, conflicting conclusions about the potential mechanism of this effect were drawn. Honegger ***et al***,[12] suggested that vitamin-E reduced the amiodarone-induced lysosomal phospholipidosis in cultured human fibroblasts by inhibiting in a dose-dependent fashion the cumulative uptake of the drug and its metabolite, desethylamiodarone, and not by an antioxidant mechanism. Both vitamin-E and silymarin-treatment combined with amiodarone decreased the number and the size of pathological lysosomes compared with amiodarone treatment alone. However, this did not entirely prevent the formation of myelin figures and electron-dense deposits.[5] Bansal ***et al***,[21] showed that the liver cells were normal, with very little necrosis (Day 21). This study concluded that the pre-treatment with vitamin-E prior to the administration of N-nitrosodiethylamine, reduced the degree of oxidative stress, although this vitamin produced only slight changes in the hepatic injury, in a time-dependent manner.

In conclusion, our study shows that vitamin-E co-administration with amiodarone led to lesser histologic changes in the parenchyma of rat liver, suggesting that vitamin-E pretreatment may play a role in the amelioration of side effects of amiodarone.
